# Adaptive Suspicious Prevention for Defending DoS Attacks in SDN-Based Convergent Networks

**DOI:** 10.1371/journal.pone.0160375

**Published:** 2016-08-05

**Authors:** Nhu-Ngoc Dao, Joongheon Kim, Minho Park, Sungrae Cho

**Affiliations:** 1 School of Computer Science and Engineering, Chung-Ang University, Seoul, South Korea; 2 Department of Information Communication, Materials, and Chemistry Convergence Technology, Soongsil University, Seoul, South Korea; University of Texas at San Antonio, UNITED STATES

## Abstract

The convergent communication network will play an important role as a single platform to unify heterogeneous networks and integrate emerging technologies and existing legacy networks. Although there have been proposed many feasible solutions, they could not become convergent frameworks since they mainly focused on converting functions between various protocols and interfaces in edge networks, and handling functions for multiple services in core networks, e.g., the Multi-protocol Label Switching (MPLS) technique. Software-defined networking (SDN), on the other hand, is expected to be the ideal future for the convergent network since it can provide a controllable, dynamic, and cost-effective network. However, SDN has an original structural vulnerability behind a lot of advantages, which is the centralized control plane. As the brains of the network, a controller manages the whole network, which is attractive to attackers. In this context, we proposes a novel solution called adaptive suspicious prevention (ASP) mechanism to protect the controller from the Denial of Service (DoS) attacks that could incapacitate an SDN. The ASP is integrated with OpenFlow protocol to detect and prevent DoS attacks effectively. Our comprehensive experimental results show that the ASP enhances the resilience of an SDN network against DoS attacks by up to 38%.

## 1 Introduction

Convergent communication network composes of multiple network architectures and technologies that supports interconnection feature over a heterogeneous network to reduce the dependence on underlying infrastructure of communication services. In recent years, the convergent communication network has been getting burdened with high requirements from users and network administrators. For instance, the users who have multimedia traffic and personal data want to transfer through higher bandwidth and more secure connections; and the network administrators require more controlling and monitoring abilities to manage the convergent networks. To satisfy all of the requirements, a next generation networking technology has emerged, known as software defined networking (SDN). One of the main advantage is that SDN is able to merge the existing network infrastructures into a new unified framework smoothly under only one requirement that all of the devices have to support an open control protocol like Openflow [1].

An SDN-based convergent network is a new approach to provide more controllable and flexible network management for better handling huge volume of data by decoupling the control functions from the forwarding hardware components [2, 3]. In an SDN-based convergent network, network control is centralized into a unified entity called the *controller* which is directly programmable. All underlying hardware elements, referred to as *switches*, take the forwarding and routing functions based on rules received from the controller. The controller acts as a framework on top of which third-party applications are deployed for a human-network interface that allows network administrators to manage the network effectively. The controllers and switches interface among them via a standard protocol. One of the most popular and widely used protocols is OpenFlow [4]. With the OpenFlow, network administrators can directly access and manipulate the forwarding functions of the underlying physical or virtual devices [1]. For convergent communications, an SDN deals with data packets using the information up to layer 4 in the header fields. Moreover, through the dynamic integration of third-party applications in network operation systems, the SDN can handle traffic flows by programmable processes to satisfy various Quality of Service (QoS) levels.

However, according to the natural feature of the centralized controller, i.e., the heart of the network in a single point, it is the Achilles heel of the SDN architecture and a potential target of flooding attacks. Therefore, three types of exploitation can be used to attack a network: the controller’s resource consumption, the controller-switch channel’s bandwidth occupation, and the switches’ flow table overload.

In a controller’s resource consumption attack, an adversary floods a huge volume of packets via switches to the controller for exceeding the capacity of the controller. This pushes the controller into an *out-of-performance* state. Therefore, the controller cannot create new flow entries for normal users in time, and this eventually makes data flows to be unstable in real-time.

The control channels between the controller and the switches can be the targets of attackers. The confidentiality and integrity of the control channel is protected by security protocols, e.g., a Secure Socket Layer, against well-known attacks such as eavesdropping or man-in-the-middle attack. However, the security protocols cannot secure the availability of the control channel against bandwidth occupation. That problem can occur when the switches are used to forward many packets to the controller in a short time period. This will eventually lead to a situation that the packets coming from normal users will be dropped at the controller’s input buffer.

Another potential security problem which makes a network malfunction is a switch flow table overload. As results of the previously described two types of attacks, i.e., consuming the controller’s resources and occupying the control channel, many flow entries continuously dispatched from the controller stack up on the switches. In some cases, the flow tables in the switches can be under overflow, even at short time. Depending on the policy, the switch should ignore the new flow entries or remove the last one in the tables. In any cases, the switches lose the warranties of services for normal users, which leads to more transmission delays. In summary, the effects of Denial of Service (DoS) attacks are serious enough to have consequences for the control channel, controller operation, and the switches’ flow table at the same time, which can negatively affect on the QoS of data services.

In order to protect SDN-based convergent networks from such attacks, we propose a novel mechanism called adaptive suspicious prevention (ASP), to protect the controller against DoS attacks. The fundamental idea is to operate the controller of an SDN-based network using specific policies to handle incoming packets differently for each user type. The specific policies are designed to decrease the influence of attacks by changing the corresponding timeouts and action parameters. To implement the idea, we have developed a mechanism based on the OpenFlow protocol. In normal times, a database of trustworthy users is built using traffic analysis. During DoS attack, all incoming unknown users are first treated as suspicious users, and their flow entries are set to very short timeouts. After that, we use a probabilistic source IP filtering technique to judiciously categorize users into the defined user types. Based on that result, the controller can treat users with exactly defined policies: update its flow entries to normal timeouts if they are trustworthy, and delete all flow entries and block them if they are malicious.

Therefore, during the attack, the controller can still serve honest users normally as well as our proposed scheme eliminates a remarkable amount of the attacking effect from malicious users. The traffic from DoS users who generate a huge volume of connections to various destinations is blocked by block entries. The useless entries of DDoS users who fake source IP addresses and establish random connections to many destinations are rapidly removed from the flow tables because they were issued with short timeout values.

Our main contributions in this paper are summarized as follows:

1. We have proposed a new algorithm, Probabilistic History based IP Filtering (PHIF), to analyze user traffic characteristics. This algorithm classifies users into five types by analyzing their numbers of connections and their average numbers of data packets per connection. The PHIF algorithm is adaptive to user traffic in real-time, even if the system is under attack.
2. We have developed a new mechanism called ASP to handle user traffic. For unknown users, we assume that they are with DDoS attacks, and apply suspicious policies, at first. According to the PHIF categorization, the controller automatically implements the corresponding policies for each recognized user type.
3. With the complete mechanism, we protect SDN-based convergent networks against both DoS and DDoS attacks. DoS attacks are canceled at the edge switches, where they intend to penetrate to get access to the network. DDoS attacks are limited in a very short timeout, and after that their flow entries are erased from the flow tables in the switches. The traffic of trusted users is still handled normally throughout the process.

This paper is organized as follows. Section 2 classifies common DoS defense strategies and particular DoS solutions on an SDN-based networks. We describe the reactive workflow inside an SDN network and break down the DoS attack process against an SDN in Section 3. Then, we propose the PHIF algorithm and ASP mechanism to resolve the problem in Section 4. Section 5 analyzes the effectiveness of useless flow entry removal and shows the effects of the proposed mechanism. We describe the performance evaluation in Section 6 to verify the correctness and effectiveness of our solution. We also discuss the limitations of the proposed mechanism in this section. Section 7 shows our conclusion about the ASP mechanism for SDN-based convergent networks and offers suggestions for future work.

## 2 Related work

### 2.1 DoS defense strategies

Many kinds of defense mechanisms can effectively combat DoS flooding attacks. The solution can come from the deployment locations or taking time approaches.

The deployment location approach separates the path from attacker to victim into three parts: source network, intermediate network, and destination network. The idea of detecting and preventing attacks on the source side is called a source-based defense mechanism. Source-based mechanisms can be deployed at client machines, gateway routers of the source network, or edge/access routers of any intermediate network. Various source-based defense techniques have been proposed to date. In [5], IP filtering detects strange addresses generated from an internal network. Another way is to monitor the different behaviors of outgoing data traffic to determine and block abnormal connections. Traffic from new users is set with low priority and limited bandwidth [6–8].

Network-based mechanisms are usually installed in intermediate networks, such as an ISP or core layer of an enterprise network. Defense techniques are based on routing requests and router identification. Because a stable set of routers participates in the core layer, a strange routing request or router can be detected easily. If they can not satisfy the verification, they will be considered malicious sources and be blocked [9–13]. A taxonomy and a conceptual cloud DDoS mitigation framework based on change point detection are also presented in [14, 15].

The idea of a destination-based mechanism is same as a source-based mechanism. One more applicable technique is the traceback, which uses packet marking or link testing to verify the source [16–18]. The same trusted data traffic model and packet filtering techniques can be found in [19, 20].

When considering the time domain, there are proactive and reactive mechanisms. Proactive mechanisms prepare defensive tools before an attack occurs. Security protocols or authentication procedures in the network layer [5] can be combined with the trusted traffic model or packet filtering techniques in the application layer [21, 22] to provide more strength against attacks. After an attack occurs, the traceback technique is also used to create a blacklist for filtering the next time. Reactive mechanisms involve defensive techniques to detect and protect against DoS during attack time. Two common mechanisms are congestion detection and traffic monitoring, as proposed in [23–25].

According to the taxonomy, our algorithm is a reactive and source-based type. It analyzes the source address of request packets arriving at the controller and uses a data statistic method in real time to divide users into five groups. After identifying user characteristics, we apply corresponding policies using flow rule modification.

### 2.2 DoS solution for SDN networks

Recently, a variety of solutions have been proposed to address security problems related to SDNs. An SDN can be an effective platform to detect and prevent attacks in IP networks (including SDN), an interesting approach that provides significant performance. Machine learning techniques are widely applied to improve detection efficiency, including neural networks, support vector machines, genetic algorithms, fuzzy logic, and Bayesian networks [26]. However, within the scope of this paper, we focus on surveying only existing solutions in which an SDN network is treated as the target of an attack. In other words, for our purposes, the SDN is a victim, not a supported tool or environment in which a security problem occurs.

Based on the controller architecture development approach, Kreutz *et al.* proposed a general design for a secure and dependable SDN control platform that helps all network components cooperate closely, interactively, and safely. However, it remains an ongoing study [27]. Recently, Shin *et al.* proposed a work named Rosemary as a network operator within a new architecture design [28]. Each application executes on a separate micro-NOS. Rosemary manages the application containment, resource monitoring, and application permission allocation to prevent cross effects between applications.

Several solutions have been proposed to tackle the vulnerability in the interface between third-party applications and the controller, such as: FortNOX [29], Fresco [30], and FermOF [31]. FortNOX is a NOX OpenFlow controller extension that provides role-based authorization and security constraint enforcement that can check for flow rules contradiction in real time, even when an adversary consciously inserts fraudulent flow rules. FortNOX works on a custom kernel to handle two types of flows, which are generated and classified by a special application, one for security requirement data and the other for the remainder. Fresco extends the work of Porras in FortNOX to introduce a framework so application developers can create and apply security services rapidly. In another approach, the FermOF system supports a set of *18* permissions between the controller and third party applications to isolate applications and check their permissions. All of that work focuses only on healing the authentication and authorization vulnerabilities among third-party applications and between applications and the controller.

Another strategy is to add new abilities to the data plane. For example, Avant-guard [32] uses two advance extensions on the data plan, called connection migration, to reduce the amount of data sent to the control plane during attack time, along with actuating triggers dispatched from the control layer that insert conditional flow rules when they detect a trigger condition based on statistics information from the data plane. To resemble Avant-guard by pushing the burden to the data plane, Kotani *et al.* proposed a packet-in message filtering mechanism to record the source addresses of requests at the switch and then forward only the first of each request to the controller [33]. Those works can reduce the volume of unnecessary data sent to the controller; however their idea seems to betray the original target of the SDN by making switches more functional and intelligent. Thus, researchers must be careful to balance between the pros and cons.

Most of those solutions strengthen the internal interactions among components in the application-controller-switch architecture to resist external attacks. Generally, they have not been proposed specially for the DoS problem, in which an SDN is the target of an attack. In our approach, we use the statistic function of the SDN to feed the PHIF filter to recognize the types of users and then apply the ASP mechanism that assigns corresponding policies for each user type to cancel DoS traffic and decrease DDoS traffic based on its characteristics. As shown in our best experiment, our algorithm can cooperate with the solutions mentioned above without any conflict because of its different approach.

## 3 Problem statement

### 3.1 Reactive work-flow inside an SDN network

An SDN network can operate in two modes: reactive and proactive. When the network operates in a proactive mode, flow entries are pre-installed in the flow tables of the switches. The header information that can be under consideration resides from layer 2 to layer 4 in an open systems interconnection (OSI) model. The proactive mode decreases the controller burden whereas it decreases flexibility in the network. In a reactive mode, the controller is in charge of managing, controlling, making decisions about routing policies, and then dispatching entries down to the switches whenever a new packet arrives [34].

**Fig 1:**
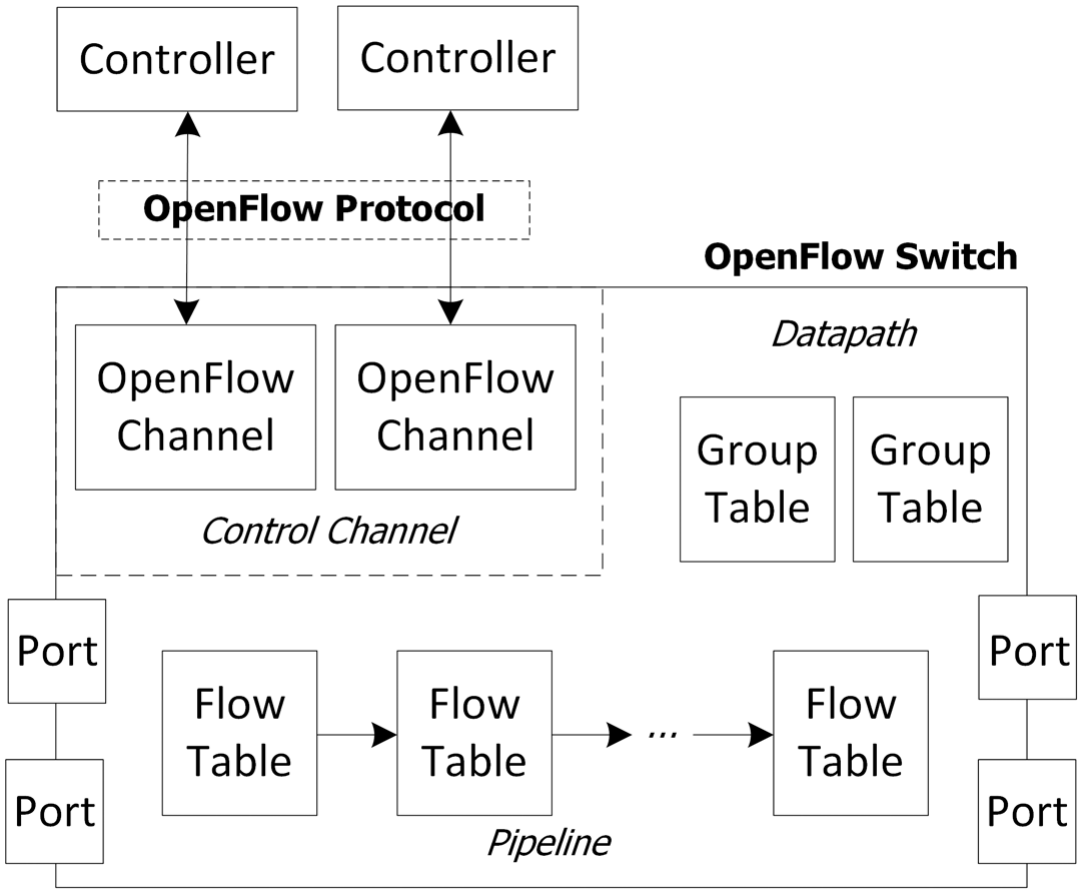
OpenFlow SDN model [36].

Fig 1 illustrates the OpenFlow SDN model. When a new packet arrives at the switch, the switch will check whether the packet header matches any entry in its flow table. If it finds a match, it will process the packet as defined in the corresponding entry. Otherwise, the switch will forward the packet to the controller. After receiving a new packet, the controller will process, calculate, and create a new flow entry for this kind of packet, which it then dispatches to the switch. The switch receives the controller message, adds the new entry into its flow table, and handles the packet as defined in the entry. Each entry has a lifetime defined by the hard_timeout and idle_timeout parameters, along with a counter of matched packets [35]. An entry expires in the number of seconds specified by the hard_timeout, or idle_timeout after a packet last hits that entry. The switch will process the next matched packets based on the corresponding entry in the flow table, as before.

### 3.2 Break-down process of DoS attacks against SDN

An attack on an SDN network includes three steps: fingerprint the target; identify the target’s characteristics that can improve the attack effect; and do the flooding attack to cause resource consumption in controller processing, bandwidth consumption in the controller-switch channel, and the switches’ flow tables to overload. When any one of these capacities is taken over, the network cannot guarantee its QoS, and malfunctions occur.

In a reactive mode, flow entries are issued by the controller only when new packets arrive at the switch. Although this mode is more flexible than proactive mode, it requires longer delays for the first packet arrival than for following ones. Using that feature, attackers can determine whether the network is an SDN by examining the response times. If the difference in response times is greater than defined threshold, we can assume that the network spent time processing for the first packet to issue a flow entry. Hence, it could be an SDN network [35].

In fact, an attacker needs no more information than that to attack an SDN network. However, because the target is resource consumption, the attack could be made more effective by knowing the lifetime parameters for the flow entries, and then appropriately controls the velocity of the flooding to correspond with the capacity of the flow table.

Typically, the next packet to arrive after a hard timeout requires the controller to take a little time to reissue a new flow entry. Therefore, to learn the hard_timeout, an attacker can send echo packets continuously and record the response times. When he detects a packet arrives with a response time equal to that of the first packet’s, the attacker can calculate the duration between those packets and approximate the hard_timeout. To calculate the idle_timeout, the attacker can use the same method but send the echo packets with ever extending durations. Whenever receiving the same response time as the first packet’s, it indicates that the idle timeout has expired, we obtain the idle_timeout value around the last extending duration.

The final step is to do the flooding attack to the network, which has two feasible approaches: DoS attack and DDoS attack. In the scope of this paper, we ignore the overload problem in a switch’s forwarding capacity to focus on an attack that makes the network malfunction. In the DoS attack method, attackers use a source IP address to send many connection establishment request packets to many random destinations to make the switches forward as many packets to the controller as possible. The controller has to process all of those packets to issue corresponding flow entries, and then the problem worsens as the switches insert all of the new, useless flow entries into their flow tables. The result is controller performance overload, occupied bandwidth in the control channel, and flow table overload. In a DDoS attack, the effect is more serious because the attack has many sources that can each behave like a DoS attacker.

**Fig 2:**
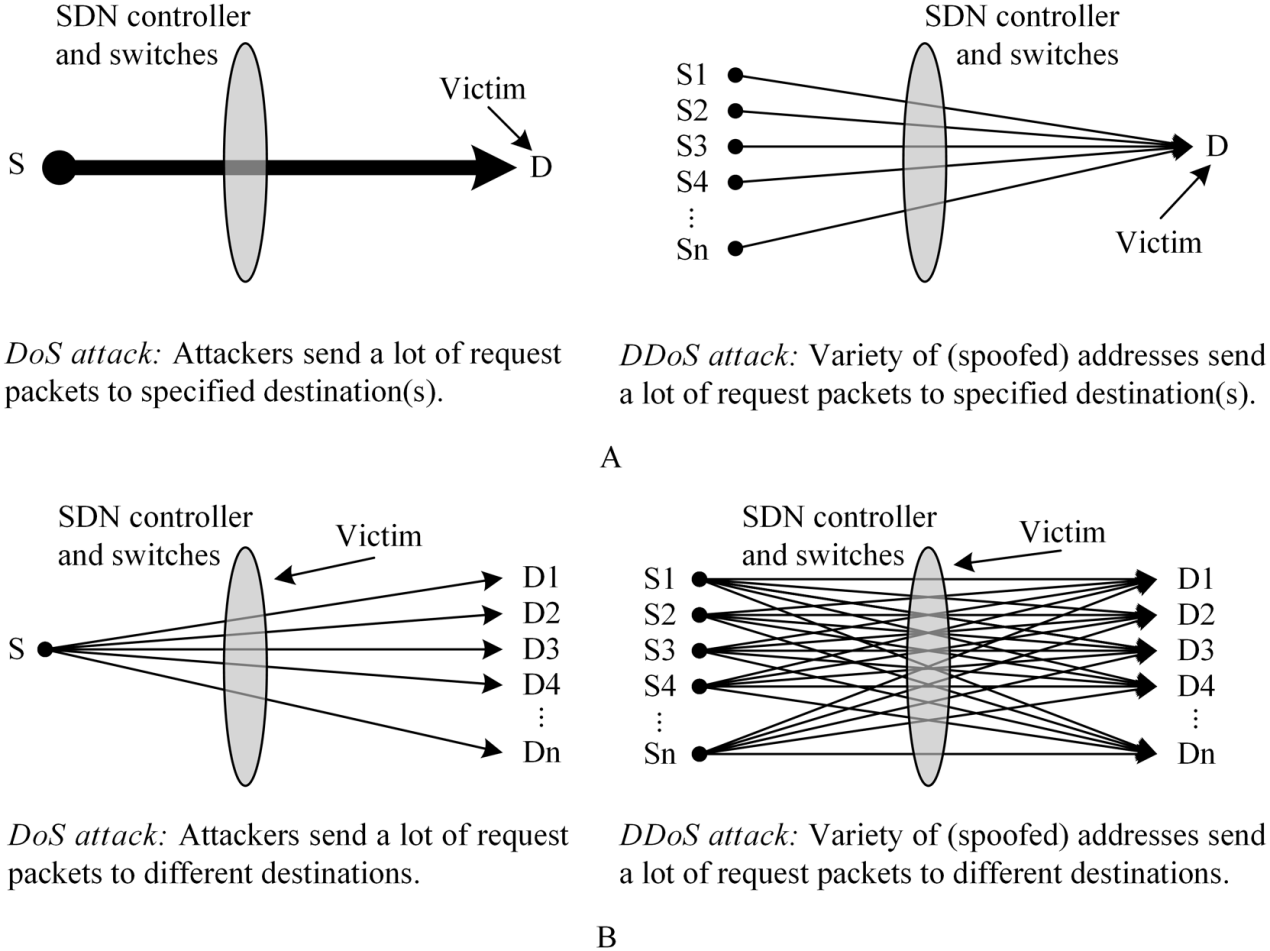
SDN as a target vs. SDN as a network environment. A) SDN as a network environment: The target is to overload specified destination(s). B) SDN as a target: The target is to overload SDN controller and switches.

Thus, we highlight different characteristics of our problem (i.e., SDN as a target) and other security-related problems on an SDN (i.e., SDN as a network environment). Fig 2 shows our comparison. In considering an SDN as a network environment, attack flows converge because the attackers intend to overload specified destination(s), not the SDN network itself. The specified destination wastes its resources to process many request packets and negotiate corresponding meaningless connections (Fig 2A). However, when an SDN is a target, attack flows diverge because the attacker’s goal is to create a huge volume of requests to overload the capacity of the SDN controller and switches. The controller and switches consume their resources to process and store useless flow entries (Fig 2B).

## 4 Adaptive suspicious prevention (ASP) mechanism

### 4.1 Mechanism design rationale

As explained in the subsection 3.2, within an SDN network, interesting targets include the controller’s resource consumption, the control channel’s bandwidth occupation, and the switches’ flow table overload. To defend against such attacks, we have developed a novel mechanism based on traffic analysis. The proposed PHIF algorithm creates an IP address database (IAD) of trusted users and recognizes users’ behavior. Based on user traffic behavior analysis, the ASP mechanism detects and protects against malicious sources during attack time.

We developed the PHIF algorithm based on the source based approach with specifics for SDN networks. To easily distinguish between the first packets of each data flow forwarded to the controller and the data packets sent from users to destinations, we call them *request packets* and *data packets*, respectively. The request packet is the packet that a switch forwards to the controller to “request” a corresponding flow entry, and the data packet is the packet transferred between user and destination. The PHIF algorithm is suitable for handling all request and data packet behaviors and can adapt to various environments. Our new defense mechanism based on the Openflow protocol (i.e., ASP mechanism) can recognize all kinds of attacks and apply corresponding policies to control them.

### 4.2 Basic assumption and environment setup

Based on the location and packet distribution characteristics, we classify users into five groups (Table 1). The user behaviors are obtained from [21].

- IAD valid user: a normal user who has a source IP address from the IAD database. Within a constant duration, an IAD valid user sends some request packets to establish connections. With each connection, the user transfers many data packets through the network.
- IAD zombie: a normal user who has a source IP address from the IAD database but has been exploited to become an attacker. An IAD zombie sends many request packets to random destination IP addresses continuously. But for each connection, the zombie transfers only a few data packets.
- Normal zombie: a new user who does not have a source IP address in the IAD database and behaves like an IAD zombie.
- Non-identified user: a new user who does not have a source IP address from the IAD database. Within a constant duration, the non-identified user sends few request packets to establish connections. We put DDoS attackers here because if we do not consider the “spoofed” feature, each source IP address from a DDoS attacker sends very few request packets. By this approach, we also classify new valid users into this group the first time they send request messages.
- New valid user: a new user who does not have a source IP address from the IAD database but does the same actions as an IAD valid user.

**Table 1:** User characteristics.

User	Source IP addresses	Request packets	Data packets	Type
IAD valid user	real&IAD	some	many	user
IAD zombie	real&IAD	many	few	DoS attacker
Normal zombie	real	many	few	DoS attacker
Non-identified user	spoofed	few	few	DDoS attacker
New valid user	real	some	many	user

In order to help recognizing the user type, we build two temporary tables in the controller to record new request packets. The valid IP table (referred to as *V* table) stores the source IP addresses of request packets from the IAD database and are forwarded from a switch in a *T*_1_ time interval. Each unique IP address has a counter *vc*_*i*_ of the number of request packets that arrive from it. It is used to calculate the average number of request packets from valid users in the *T*_1_ time interval. The new IP table (called *N* table) stores the source IP addresses of request packets that are not from the IAD database and are forwarded to the controller from a switch in a *T*_2_ time interval. Each IP address has a counter *nc*_*i*_ to track the number of packets that arrive from it.

### 4.3 Probabilistic History-based IP Filtering

As we explained above, normal user behavior is to send some request packets in a constant duration. For each connection, the user transfers many data packets. Therefore, in this section we will explore how many packets are sent by each user type. Let the range of the number of request packets be [*k*_1_, *k*_2_] and the minimum number of data packets per connection be *n*. As shown in [21], the value of *n* is not less than 3.

In a normal traffic environment, we can assume that the incoming request packets have a Poisson distribution with parameter *λ* equal to the average number of request packets arriving. Fortunately, the valid IP table can represent normal traffic conditions even if an attack occurs because we know exactly how many normal request packets arrive in a *T*_1_ time interval. Hence, the average number of request packets is

$$\begin{array}{c} \lambda =\frac{\sum_{i=1}^{k}vc_{i}}{k} \end{array}$$ (1)

where *k* is the number of IPs in the valid IP table.

We define *x* (percent) as the tolerance for accuracy in determining of request packets. Because the probability mass function of incoming request packets is symmetrical by parameter *λ*, we can determine *k*_1_ and *k*_2_ as

$$k_{1}=\lambda -i$$ (2)

$$k_{2}=\lambda +i$$ (3)

where *i* satisfies

$$\begin{array}{c} \begin{array}{c} x=\sum_{j=0}^{\lambda +i}\frac{e^{-\lambda }\lambda^{j}}{j!}-\sum_{j=0}^{\lambda -i}\frac{e^{-\lambda }\lambda^{j}}{j!}\\ \Leftrightarrow x=\sum_{j=0}^{2i}\frac{e^{-\lambda }\lambda^{j}}{j!} \end{array} \end{array}$$ (4)

The range of [*k*_1_, *k*_2_] automatically changes based on the probabilistic model every *T*_1_ seconds according to the real traffic environment (Algorithm 1).

**Algorithm 1** Probabilistic History-based IP Filtering

1: *x* ← the acceptance percentage;

2: Initialize *k*_1_;

3: Initialize *k*_2_;

4: **loop**

5:  *λ* = avg(*vc*_*i*_);

6:  *j* = 0;

7:  **while** *poisscdf*(*j*, *λ*)<*x* **do**

8:   *j* + +;

9:  **end while**

10:  $k_{1}=\lambda -\left(\frac{j}{2}+\frac{1}{2}\right)$;

11:  $k_{2}=\lambda +\left(\frac{j}{2}+\frac{1}{2}\right)$;

12:  countdown(*T*_1_);

13: **end loop**

To determine the minimum number of data packets per connection, *n*, the controller sends statistic request messages to each switch with the matched field equal to all IP addresses in the *V* table. Based on the collected packet_count parameters from the response messages, we use the minimum packet_count value for each IP address to derive the value of *n*

$$\begin{array}{c} n=\max(3,\min_{\forall i\in V}\left(\text{packet}\_{\text{count}}_{i}\right)) \end{array}$$ (5)

where *packet*_*count*_*i*_ is the minimum packet_count value of the *i-th* IP address.

### 4.4 Adaptive suspicious prevention mechanism

The ASP mechanism covers two network phases: normal time and attack time.

In normal time, we apply the PHIF algorithm to build the IAD database. The rule is that whenever an IP address has a number of request packets in the range of [*k*_1_, *k*_2_] in T1 seconds, it will be added to the IAD database.

In attack time, the consideration has to be more particular. Two main characteristics differentiate between the traffic of a normal user and that of an attacker. One is about request packets: most normal users establish a number of connections in the range of [*k*_1_, *k*_2_], but attackers request fewer than k1 packets (DDoS type) or more than k2 packets (DoS type), see Fig 2b. The other factor is about data packets: normal users frequently transfer more than *n* packets per connection, whereas attackers usually transfer fewer than *n* packets. Based on those differences, our ASP mechanism analyzes incoming traffic to recognize user types and apply corresponding policies. The mechanism runs as an application in the controller.

**Algorithm 2** Adaptive suspicious prevention mechanism

1: *D* ← the IP Address Database IAD;

2: *N* ← the new IP table set;

3: *V* ← the valid IP table set;

4: **loop**

5:  **if** source IP ∈ *D* **then**

6:   normal processing;

7:   update its *vc*_*i*_ in V table;

8:   **if** *vc*_*i*_ ≥ *k*_2_ **then**

9:    request statistic of its flow entries (called *s*);

10:    **if** *s* ≤ *n* **then** {//*IAD zombie*}

11:     block its IP address by setting hard_timeout to *h* seconds;

12:     remove all of its previous flow entries;

13:     remove it from D database;

14:    **else**

15:     do nothing;

16:    **end if**

17:   **else**

18:    do nothing;

19:   **end if**

20:  **else** {//*non-identified user*}

21:   add a corresponding flow entry with short idle_timeout to *d* seconds;

22:   update its *nc*_*i*_ counter in N table;

23:   **if** *nc*_*i*_ ≥ *k*_1_ **then**

24:    request average number of packet_count of its flow entries, *s*;

25:    **if** *s* > *n* **then** {//*new valid user*}

26:     update its existing flow entries with normal idle_timeout;

27:     move it from N table to V table;

28:     add it to D database;

29:    **else** {//*normal zombie*}

30:     block its IP address by setting hard_timeout to *h* seconds;

31:     remove all of its previous flow entries;

32:    **end if**

33:   **else**

34:    do nothing;

35:   **end if**

36:  **end if**

37: **end loop**

**Fig 3:**
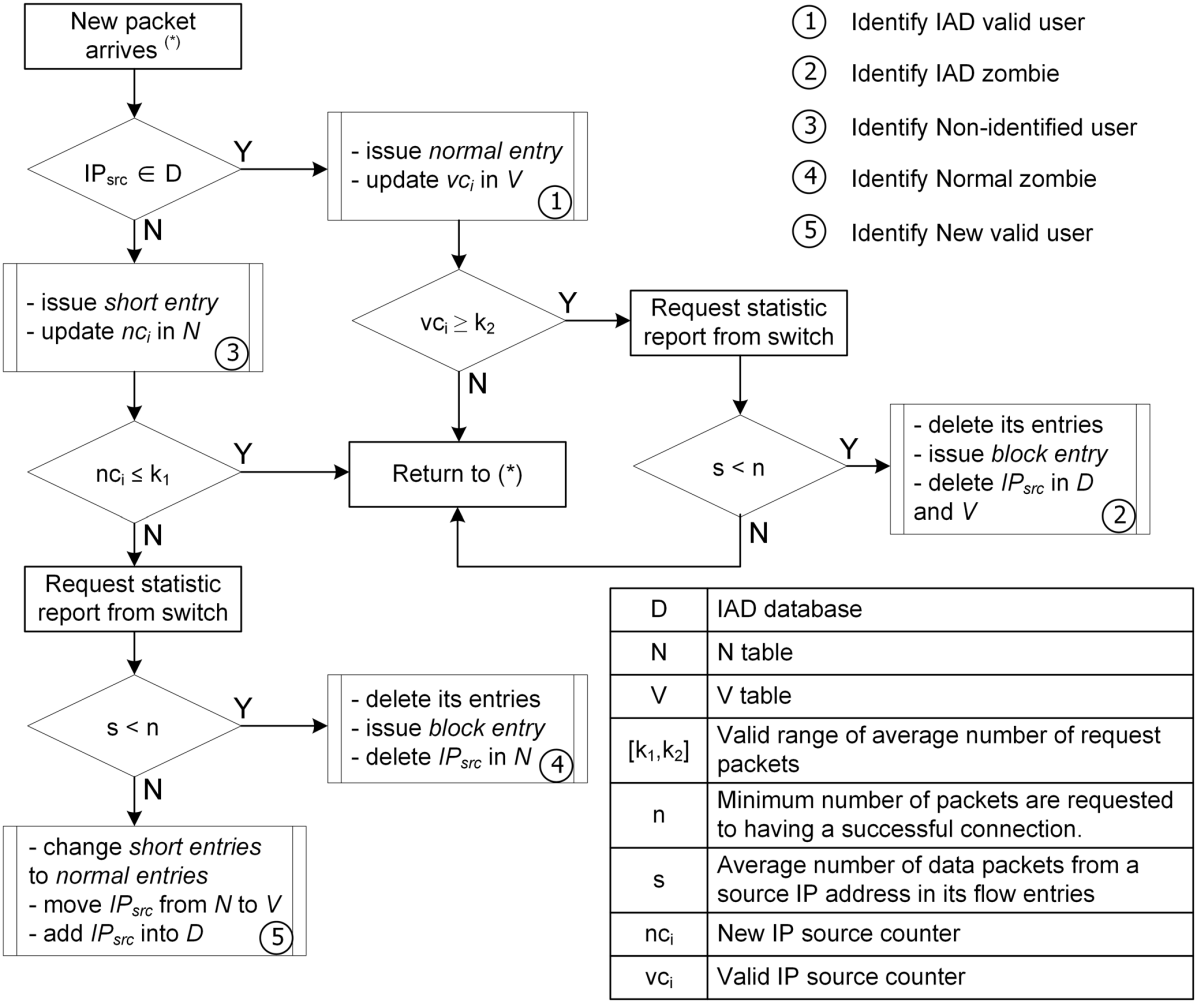
Flow diagram of request packet processing in the controller.

Consider a flow diagram in Fig 3. Whenever a new request packet arrives at the controller, the controller checks whether the source IP address belongs to the IAD database or not. If it does, we can determine that the packet comes from a trusted user. The controller processes it normally and updates the source IP address in the *V* table, increasing its *vc*_*i*_ counter by *1*.

However, we also need to check its request packet behavior. If its *vc*_*i*_ exceeds *k*_2_, it might be an abnormal sign and require a data traffic characteristics analysis. To do that, the controller issues an OFPMP_FLOW statistic request message to the switches for the packet_count of the source IP address. Then, the controller calculates the average packet number *s*. If *s* is smaller than *n*, the source IP address is identified as an IAD zombie. The policy for an IAD zombie is: (1) the controller issues an OFPT_FLOW_MOD message with the OFPFC_DELETE value installed as the command parameter to force the switches to delete all flow entries from that source IP address. (2) The controller sets a block rule for the source IP address in the switches’ flow table using an OFPT_FLOW_MOD message with a hard_timeout parameter of *h* seconds [7]. (3) The source IP address is deleted from the IAD database. Therefore, all new traffic from this source IP address will be dropped for the next *h* seconds. After that, it will be treated as a new user (see *Algorithm 2, line 5–19*).

If the source IP address does not belong to the IAD database, we first classify it as a non-identified user. The policy for a non-identified user is as follows: (1) the controller issues an OFPT_FLOW_MOD message with the idle_timeout parameter set to *d* seconds, which is much smaller than the normal idle_timeout parameter. (2) The source IP address is updated into the *N* table for tracking and its *nc*_*i*_ counter increases by *1* (see *Algorithm 2, line 20–22*).

When *nc*_*i*_ reaches *k*_1_, we analyze its data traffic characteristics by calculating the average number of packets *s*. If *s* is greater than *n*, the source address has established and transmitted real data connections. In other words, it is an honest user and must be trusted. The policy for a new valid user is: (1) the controller issues an OFPT_FLOW_MOD message with the command parameter OFPFC_MODIFY, and the idle_timeout is installed to the normal value, which affects all its flow entries. (2) The source IP address is moved from the *N* table to the *V* table and is stored in the IAD database as a new valid user (see *Algorithm 2, line 23–28*).

**Fig 4:**
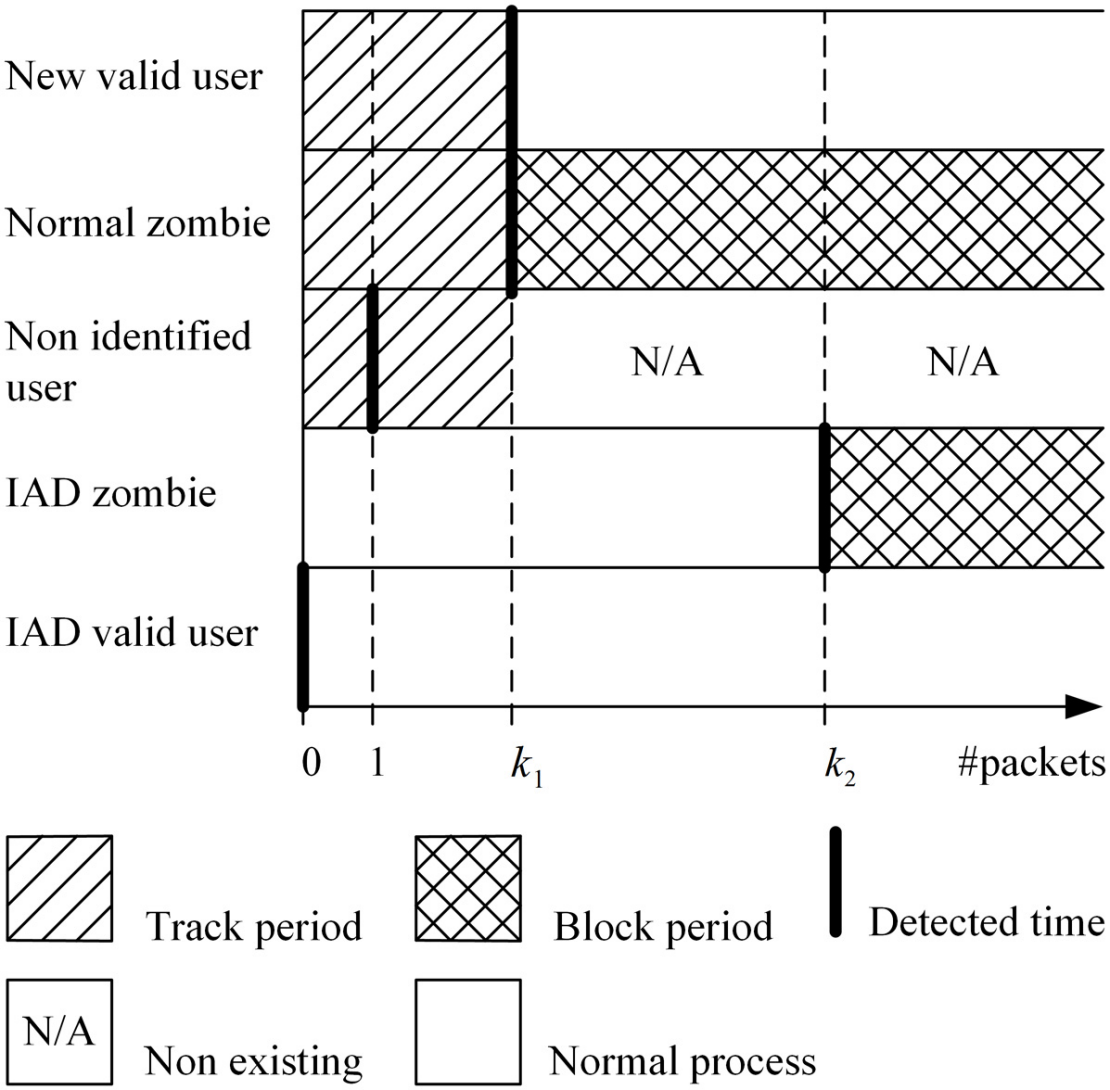
The detect time of user types.

In contrast, if *s* is smaller than *n*, the source address is considered to be a normal zombie. The policy for a normal zombie is as follows: (1) the controller issues modified messages to delete all its existing entries in the flow table. (2) The controller generates a block rule for the source address during *h* seconds, as in the rule for IAD zombies (see *Algorithm 2, line 29–31*). Thus, the ASP detects all user types and uses suitable policies for each one (Fig 4).

## 5 Effects of the ASP mechanism

We evaluated the effect of the ASP mechanism by analyzing the number of flow entries in the switches. A DoS attack floods new packets to the SDN network to occupy the control bandwidth, consume the controller’s resources, and overload the switches’ flow tables. These are sequential effects. The number of flow entries in the switches (represented for the flow tables’ overload) is directly proportional to the number of forwarded new packets that arrive at the controller (represented for the control channel occupation) and the number of entries that the controller generates (represented for the controller’s resource consumption).

To analyze the number of flow entries in the switches, we used the work flow of normal network operation and network operation within the ASP mechanism given in Subsections 3.1 and 4.4, respectively. Let the speed of new incoming data flows from IAD valid users, new valid users, and non-identified users be *d*_1_, *d*_2_, and *d*_3_ flows per second, respectively. The speed of new incoming packets from IAD zombies and normal zombies are $n_{4}\bar{d_{4}}$ and $n_{5}\bar{d_{5}}$, respectively, where *n*_*i*_ is the number of clients and $\bar{d_{i}}$ is the average number of new incoming flows from each client. The value of (hard_timeout, idle_timeout) for normal entry, short entry, and block entry are given by (*h*, *e*_*n*_), (*h*, *e*_*s*_), and (h*, 0), respectively.

According to the description in Subsection 3.1, the controller processes all forwarded packets arriving from the switches, then issues corresponding flow entries for the switches to insert into their flow tables. We assume that the normal flows transfer data continuously until the hard_timeout expires and that faked flows transfer very few packets until the idle_timeout expires. Therefore, the mean of the number of flow entries is

$$\begin{array}{c} {\bar{F}}_{1}≅d_{1}h+d_{2}h+d_{3}e_{n}+n_{4}\bar{d_{4}}e_{n}+n_{5}\bar{d_{5}}e_{n} \end{array}$$ (6)

Following the description of the ASP mechanism given above, the system applies a separate policy to each type of user. IAD valid users and new valid users have normal flows that transfer data until the hard_timeout for normal entry expires. Each non-identified user transfers very few packets, and its corresponding flow entries expire after the short entry idle_timeout. New incoming flows from each IAD zombie or normal zombie are dropped immediately by the block entry after the network detects its behavior. Because the analysis is performed within a large volume of data, the effect of each IAD zombie and normal zombie before it is detected is very small, which we denote by the function $\delta_{t}\left(n_{4}\bar{d_{4}},n_{5}\bar{d_{5}}\right)$. The mean of the number of flow entries within the ASP mechanism is given by

$$\begin{array}{c} {\bar{F}}_{2}≅d_{1}h+d_{2}h+d_{3}e_{s}+n_{4}+n_{5}+\delta_{t}\left(n_{4}\bar{d_{4}},n_{5}\bar{d_{5}}\right) \end{array}$$ (7)

As shown by a comparison between Eqs (6) and (7), the portion related to IAD valid users and new valid users is the same. The ASP mechanism has a special effect in DoS attacks in terms of the reduction of the number of entries by $n_{4}\left(\bar{d_{4}}e_{n}-1\right)+n_{5}\left(\bar{d_{5}}e_{n}-1\right)-\delta_{t}\left(n_{4}\bar{d_{4}},n_{5}\bar{d_{5}}\right)$. In a DDoS attack, the number of entries decreases by a volume of *d*_3_(*e*_*n*_ − *e*_*s*_). Because the *n*_*i*_ and $\bar{d_{i}}$ are objective, for improving the performance of the solution, (i) the short entry idle_timeout, *e*_*s*_, should be small as much as possible; and (ii) the normal idle_timeout, *e*_*n*_, should be large as much as possible while maintaining some transmission constraints (e.g., the *e*_*s*_ must greater than the propagation delay, a large *e*_*n*_ will cause the flow table in the switches to be overloaded). Therefore we have a plan to optimize these parameters to achieve an effective performance in our future research direction.

## 6 Performance evaluation

### 6.1 Network topology

**Fig 5:**
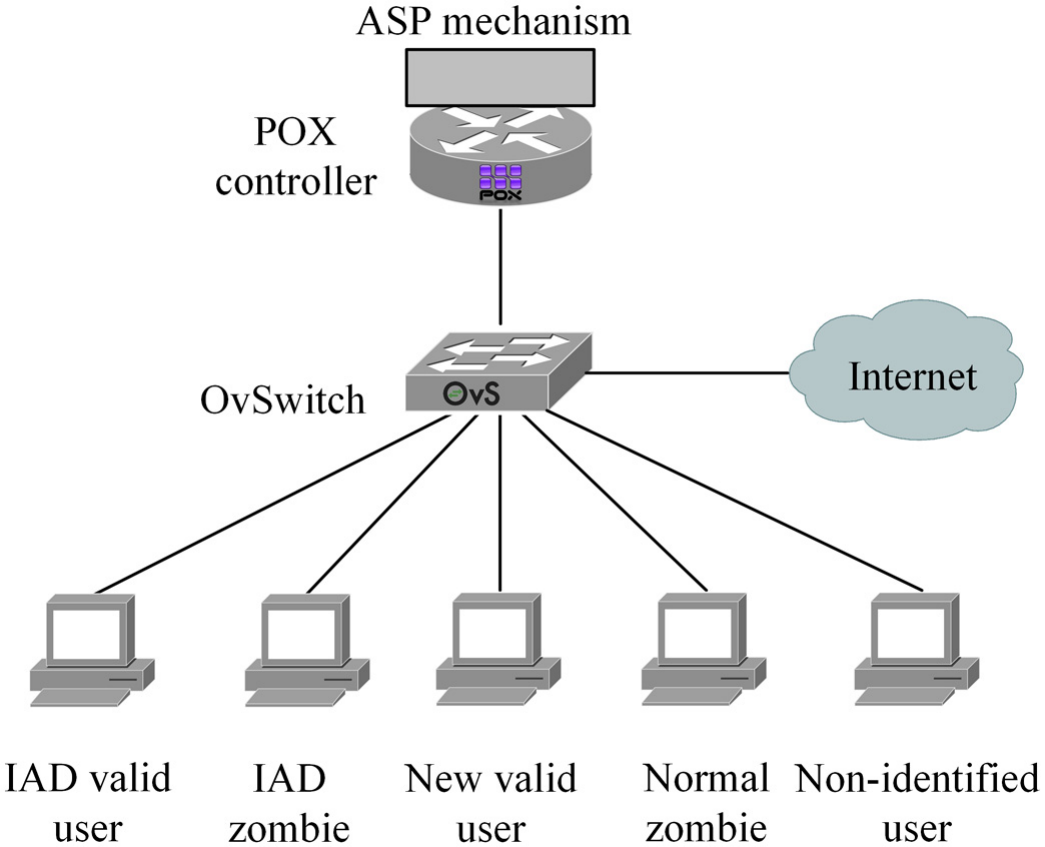
Emulation topology.

To verify the performance of the algorithm, we built a network topology consisting of a POX controller, an OpenvSwitch, and five PC clients (representing the five user types). The network topology was emulated by Mininet 2.1.0 on the Virtualbox 4.3.20 framework (Fig 5). By default, Mininet cannot support this emulation scenario and topology. To implement the emulation network, we extended the default functions of Mininet to meet our requirements. We deployed the controller as a RemoteController to be ready to listen to the modified POX controller.

c0 = net.addController(’c0’, controller = RemoteController)

Mininet VM reserves one network adapter for the Host-only Adapter configuration for administration purposes. The remainder are integrated into OpenvSwitch as its ports.

#### 6.1.1 Normal routing controller

**Fig 6:**
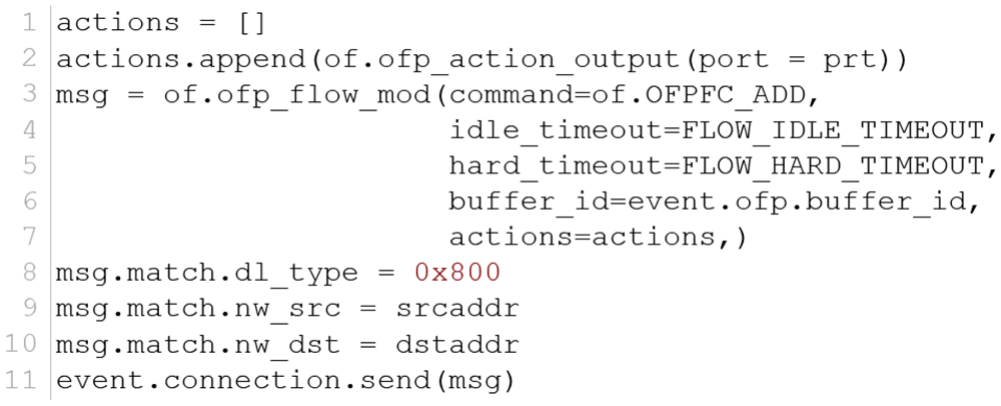
Normal routing controller actions.

Basically, layer 3 devices route packets based on the source and destination IP addresses. The default example of an l3_learning controller considers not only the layer 3 addresses but also all the information in the header of the incoming packet. Therefore, we rebuilt a new layer 3 routing controller, which runs as a standard router to issue clearer flow entries for OpenvSwitch. ARP and echo packets are forwarded without any policy. The TCP/IP packets are tracked to create reasonable entries in the flow tables (Fig 6).

#### 6.1.2 Internet access

Internet access connection is supported under the NAT network function configuration of Virtualbox. The configured adapter integrates into OpenvSwitch directly; it acts as a gateway for the SDN network to contact the outside. The NAT network adapter could provide some helpful applications such as DHCP, NAT services, and the IPv6 protocol. All network clients should belong to the same subnet of the adapter; the IP addresses are auto-configured by the DHCP service or can be configured manually. We chose the 10.0.0.0/24 private IP address class for PC clients.

#### 6.1.3 Independent OS clients

The client VMs connect to OpenvSwitch through internal network adapters. The adapters integrate into the switch as its ports. Each client VM requires separate configuration of its corresponding adapter using the following commands:

$vboxmanage modifyvm Mininet –nic1 intnet
$vboxmanage modifyvm Mininet –intnet1 “intnet01”
$vboxmanage modifyvm Mininet –nicpromisc1 allow-all

Based on those connections, the switch can interface with real independent OS client VMs and access the Internet.

_intf = Intf(’eth1’, node = s1)

Because the clients do not depend on SDN topology installation, they could operate on any kind of full featured OS, such as Windows, Linux, and even Mac or Android. Based on the extension, we installed Windows 10 and Ubuntu 14.10 in the PC clients as requirements.

#### 6.1.4 Automatic flow logging

Mininet provides some commands to manually check the status of the switches and controller. It might be helpful for learning but is inadequate for research activities because we would like to receive information systematically and exactly. In the emulation network, we developed a flow logging function that helps to periodically export the main status of flow entries in the switches and flow requests arriving at the controller. The statistic request messages are sent to the switches at defined intervals.

body = of.ofp_aggregate_stats_request()
body = of.ofp_flow_stats_request()

The handle functions are programmed to catch statistic report messages. Collected data are processed to export useful information into logging files.

def _handle_AggregateFlowStatsReceived (self, event)
def _handle_FlowStatsReceived (self, event)

### 6.2 An example analysis

We defined three types of flow entry:

- Short entry: has hard_timeout equal to *60* seconds and idle_timeout equal to *5* seconds.
- Normal entry: has hard_timeout equal to *60* seconds and idle_timeout equal to *10* seconds.
- Block entry: has hard_timeout equal to *3600* seconds.

To verify the work flow of the algorithm when processing each type of incoming packet at the controller, we implemented a scenario that includes five clients, one for each user type. The configurations for the five PC clients are given in Table 2. We pre-installed IP addresses *10.0.0.1, 10.0.0.2* into the IAD database as IAD valid users who were found and stored in the normal traffic environment. The role of each PC client is described as follow:

- IAD valid user (10.0.0.1/32) acts as a valid user all time.
- IAD zombie (10.0.0.2/32) changes from a valid user to a DoS attacker.
- Normal zombie (10.0.0.3/32) acts as a DoS attacker all time.
- Non-identified user (10.0.0.11/24) acts as a DDoS attacker who generates random IP sources to flood faked packets into the network.
- New valid user (10.0.0.5/32) acts as a valid user who uses the network for the first time.

**Table 2:** PC clients configurations.

PC client	OS	IP address	Actions
IAD valid user	Windows 10 pro	10.0.0.1/32	Continuously sends IP packets to valid destinations on the Internet.
IAD zombie	Windows 10 pro	10.0.0.2/32	Sends IP packets to various non-valid destinations. For each destination, the IAD zombie sends only 1 packet. Waiting time between messages is 200ms.
Normal zombie	Windows 10 pro	10.0.0.3/32	Sends IP packets to various non-valid destinations. For each destination, the normal zombie sends only 1 packet. Waiting time between messages is 200ms.
Non-identified user	Ubuntu 14.10	10.0.0.11/24	Using the DDOSIM tool [37], continuously sends IP packets to webserver 10.0.0.101. The source IP address was spoofed randomly in the range of 10.0.0.11/24. Waiting time between requests is 200ms.
New valid user	Windows 10 pro	10.0.0.5/32	Continuously sends IP packets to valid destinations on the Internet.

All PC clients are run at the same time. After that, we record the flow table status in two scenarios: using a normal POX controller and using an ASP-enabled controller.

The log file recorded in the ASP-enabled controller indicate that the IP addresses *10.0.0.3*, *10.0.0.2* were detected as a normal zombie and an IAD zombie after *5* and *10* seconds, respectively. The IP address *10.0.0.5* was recognized as a new valid user after *50* seconds.

**Fig 7:**
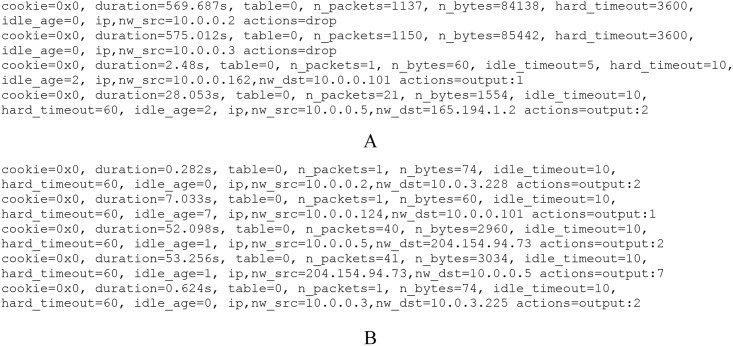
Flow tables in the experimental OpenvSwitch. A) Under the control of the ASP-enabled controller. B) Under the control of the normal POX controller.

Fig 7A represents the flow table in the switch under the control of the ASP-enabled controller. The first two entries are block entries for the IAD zombie (*10.0.0.2*) and normal zombie (*10.0.0.3*). The *action* parameter was set to “drop”, and the hard_timeouts are *3,600* seconds. By duration *570s*, more than *1,000* packets (i.e., equivalent to *1,000* meaningless data flows) were dropped from each zombie. The fourth entry shows that the source IP address *10.0.0.5* has hard_timeout *60s* and idle_timeout *10s*, which means that the address was treated as a valid user (new valid user). Other IP addresses have hard_timeout *10s* and idle_timeout *5s* as non-identified users (e.g., *10.0.0.162* in *10.0.0.11/24* class that is generated by non-identified user).

In contrast, Fig 7B describes the switches’ flow table under the control of the normal controller. All flow entries have the same value of hard_timeout *60s* and idle_timeout *10s*, even for the zombies *10.0.0.2* and *10.0.0.3*.

The ASP-enabled controller blocks malicious traffic from the DoS attackers (IAD zombie and normal zombie); the network no longer has to process their request and data packets. Therefore, the ASP decreases the controller-switch bandwidth occupation and the number of useless flow entries. Besides that, traffic from the DDoS attackers received short timeout entries, so it will be removed very soon. However, the existing (IAD valid user) and new valid users (new valid user) were still served as normal. In the mechanism, we manually configured the timeout parameters of short entry, normal entry, and block entry. According to particular environments and networking requirements, we can tune the timeouts for better performance.

### 6.3 Performance evaluation

To evaluate the algorithm’s performance, we pushed a huge volume of input data into the network. Based on the experienced topology, we changed the emulation characteristics in the PC clients. In the IAD valid user and new valid user’s PCs, we used half of the 100,000 captured packets in the CAIDA Anonymized Internet Traces Dataset [38] for each (Table 3), see S1 Dataset. First, to build the IAD table, we operated the IAD valid user’s PC to inject packets into the network. Based on the algorithm, the controller recorded a list of source IP addresses into the IAD table as IAD valid users. To emulate the IAD zombie data, we extracted 100 source IP addresses from the IAD table and changed their behavior. Each IAD zombie address sent one IP packet to all 100 random destinations. For normal zombies, we also chose 100 new source IP addresses and assigned the same behaviors as for the IAD zombies. We sped the non-identified users’ data by reducing the wait time between requests to 10ms and generating 10,000 packets to the network (Table 2). The values of (hard_timeout, idle_timeout) for normal entry, short entry, and block entry are given as *(60, 5)*, *(60, 1)*, and *(3600, 0)*, respectively. We compare the numerical results among the proposed ASP mechanism, proactive source based filtering (SBF), reactive data flow analysis (DFA), and a normal POX controller.

**Table 3:** A part of CAIDA Anonymized Internet Traces Dataset.

Total number of IP packets	100,000
Total number of IP addresses	11,011
Number of flows	5,957
Average number of packets/flow	14.58
Number of flows of the IP addresses with fewer than 3 flows or 3 packets/flow	5,233
Number of flows of the IP addresses with more than 3 flows and 3 packets/flow	724
Number of IP addresses with more than 3 packets/flow	5,037
Number of IP addresses with more than 3 flows and more than 3 packets/flow	118
Number of IP addresses with more than 3 flows and fewer than 6 flows with more than 3 packets/flow	97

In proactive SBF, the filter sets a threshold for request packets arriving at the controller. When the volume of request packets is below the threshold, the controller processes all request packets without any changes. If the volume of request packets grows over the threshold, the controller prioritizes request packets from IAD users first and drops other request packets that exceed the threshold (referred to [6–8]).

Within the reactive DFA, in the first seconds, the controller does not have any knowledge about the forwarded data; and thus it handles all request packets as normal and starts to learn about user data behavior. Using its data traffic analysis over time (e.g., analyzing the number of data packets in each flow of the users), the controller builds its own blacklist of sources that generate abnormal traffic. Based on this blacklist, the controller is able to block most of DoS attack flows (referred to [21]).

**Fig 8:**
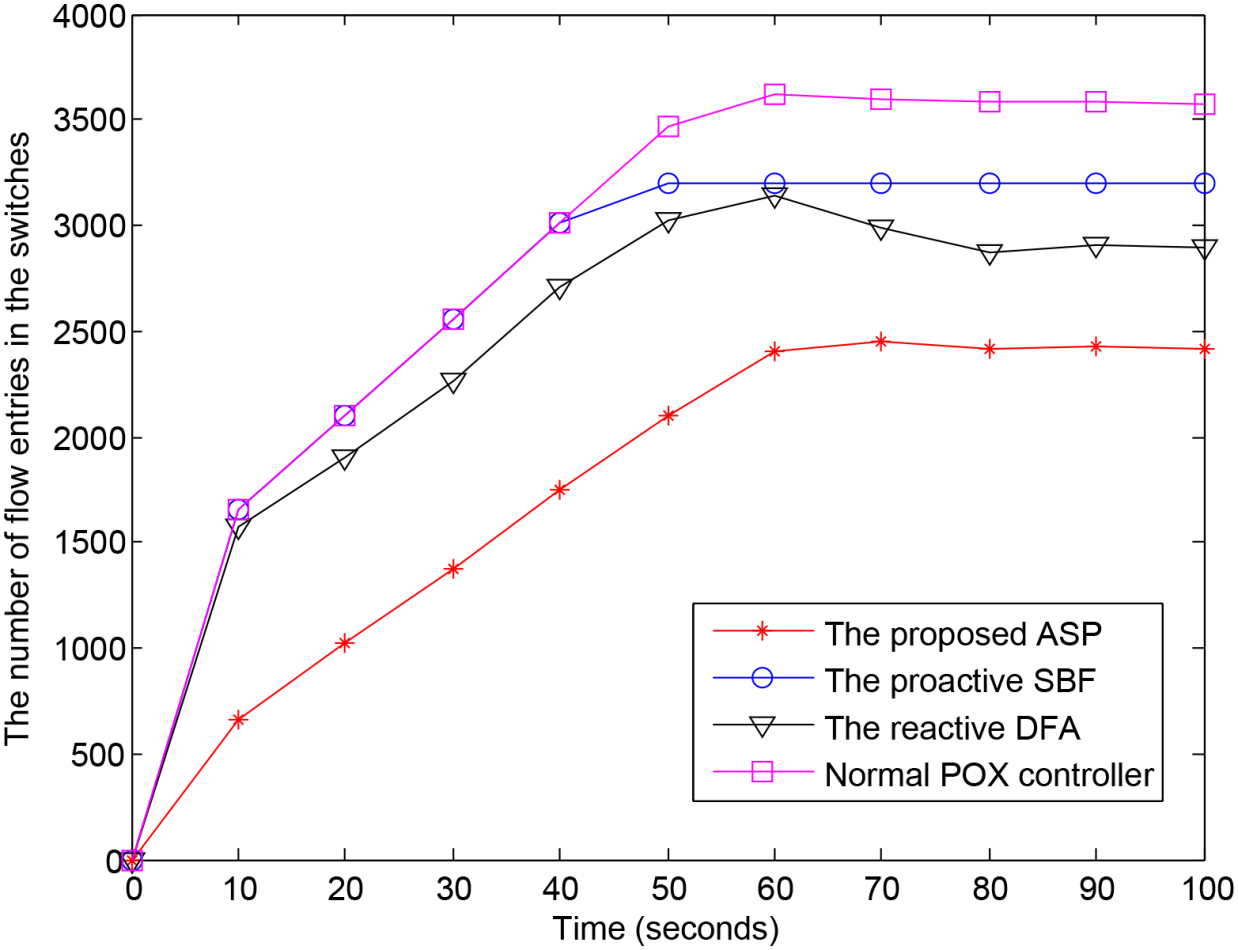
Number of flow entries in the switch.

Fig 8 compares the number of flow entries in the OpenvSwitch among the 4 experimental mechanisms. In the first second, the number of flow entries in the 4 controllers are almost the same. Because the idle_timeout of the short entry is set to 1s in the ASP mechanism, from second 2, its number of flow entries in the switch increases more slowly than that in the other mechanisms because of the volume of flow entries for non-identified users and DDoS attackers being erased from the flow tables. Also, some source IP addresses from IAD zombies and normal zombies are detected and then blocked by the corresponding block entries. With the normal POX controller, the number of flow entries in the switch increases very quickly from seconds 1 to 5. After second 5, the growth increases more slowly because the idle_timeout for normal entry starts to take effect. Some flow entries expire and are erased from the flow table. From second 60 (equal to the hard_timeout value), the number of flow entries in the switch has become stable. The number of erased flow entries and newly issued flow entries are approximate values. The gap between the number of flow entries in the switch within the ASP-enabled controller and the normal POX controller comes from the erased short entries for non-identified users and DDoS attackers and the block entries from IAD zombies and normal zombies.

In proactive SBF, the filter sets a threshold for request packets arriving at the controller. When the volume of request packets is below the threshold, the controller processes all request packets as normal. After second 50, the volume of request packets reaches the threshold; therefore, the controller prioritizes request packets from IAD users first and drops other request packets that exceed the threshold. Within the reactive DFA, in the first seconds, the controller does not have any knowledge about the forwarded data; it thus handles all request packets as normal and starts to learn about user data behavior. Over time, using its data traffic analysis, the controller builds its own blacklist of sources that generate abnormal traffic. Based on this blacklist, the controller can block almost DoS attack flows. However, the controller cannot adapt to issue appropriate flow entries for DDoS attacks.

**Fig 9:**
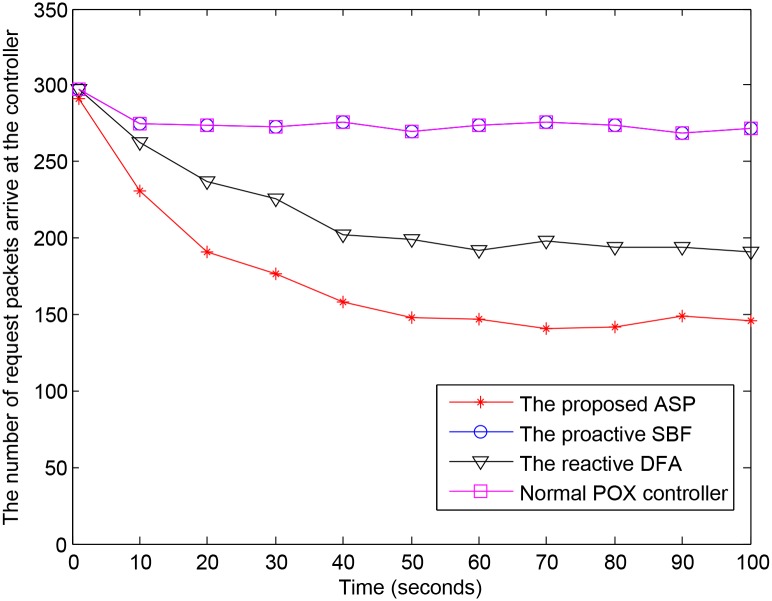
Number of request packets arriving at the controller.

Fig 9 shows the number of packets that a switch forwards to the controller. In the first second, most new packets coming to the switch are forwarded to the controller; there is no significant difference among the experimental mechanisms. During some early seconds, the number of packets arriving at the ASP-enabled controller is even greater than at the other controllers because the ASP-enabled controller assigns short entries for the first 3 flows of new valid users, but they expire very soon, and the switch has to re-forward request packets to the ASP-enabled controller. However in the next seconds, when the ASP-enabled controller detects IAD zombies and normal zombies, it generates corresponding block entries for them. After that, all of their incoming packets are dropped at the switch, and no more of their request packets are forwarded to the ASP-enabled controller. The result is that the number of packets arriving at the controller decreases since all the IAD zombies and normal zombies are blocked. The proactive SBF does not reduce the number of request packets arriving at the controller, and it thus has the same level as the normal POX controller. On the other hand, the reactive DFA obtains better results than the proactive SBF. However, the DFA reacts only after making a data analysis, whereas the ASP also uses the IAD trusted database, request packet behavior analysis, and the ASP workflow to reduce its reaction time and improve its quality of detection. Therefore the reactive DFA cannot achieve performance as high as that of ASP.

Table 4 presents a numerical result of the bandwidth occupation in the switches-controller channels. The time records include initial period in 10 seconds (i.e., the ASP starts with an assumption that all arriving unknown users are DDoS zombies), middle period in 50 seconds (i.e., the ASP is collecting and analysing report data from the switches to issues corresponding policies for each user type), and stable period (i.e., the ASP operation obtains stable state when the number of arriving users and the number of processing users are equivalent). When the system achieves stable state, the proposed ASP decreases the occupied bandwidth down to 46.55%, 25.21%, and 46.55% compared to the proactive SBF, reactive DFA, and POX controller, respectively.

**Table 4:** Bandwidth occupation in the switches-controller channels.

Time	Proposed ASP (Kbps)	Proactive SBF (Kbps)	Reactive DFA (Kbps)	POX controller (Kbps)
Initial period	1,263	1,234	1,234	1,234
Middle period	786	1,140	963	1,140
Stable period	605	1,132	809	1,132

The effectiveness of the ASP mechanism is shown in Table 5. The average number of flow entries in the switch within the ASP-enabled controller is reduced up to 38.23% compared with the normal POX controller. The average number of request packets arriving at the ASP-enabled controller per second is reduced up to 36.17%.

**Table 5:** Comparison between the experimental controllers.

Statistics	Proposed ASP	Proactive SBF	Reactive DFA	POX controller
Average number of flow entries	1,899	2,853 (↓ 33.40%)	2,626 (↓ 27.64%)	3,076 (↓ 38.23%)
Average number of request packets per second	176	275 (↓ 36.17%)	217 (↓ 19.15%)	275 (↓ 36.17%)

### 6.4 Limitation and discussion

Through our experiment and analysis, we have realized that our proposed ASP mechanism still has some limitations. Because the controller has to maintain the tables of source IP addresses and seek an equivalent entry whenever a new forwarded packet arrives, it needs more time for packet processing. Theoretically, this problem leads to greater delay time for the network to handle its traffic. However, in SDN network, the controller can be deployed on high performance server, the additional delay time is not significant. Moreover, we can reduce the effects of the delay time by using an advanced search algorithm to improve the search time in the table.

Second, the ASP mechanism generates overhead when the controller requests trigger reports (to verify the data traffic behavior of a specific user) and periodic reports (to calculate the minimum number of packets per successful connection—parameter *n*) from the switches. Hence, we must to balance between report period and overhead to get the optimal benefit.

The third is about the assumption of user behaviors. In this paper, we start by using the result in [21] as our initial assumption of user behaviors. However, the dependence to this work is not considerable because we have compensated for this shortcoming to reduce the case of false negative detection by using the proposed PHIF mechanism that adapts with the variance of different network environments and the novel ASP workflow that defines the non-identified user type to first assign a short entry. After that, we checked their continuous flow requests and data traffic behavior to determine the exact type of user.

The last limitation is that our mechanism has special effectiveness against DoS attacks, but for DDoS attacks, the mechanism reduces only part of the effect by using the short timeout. Thus, the ASP is not a complete solution for DDoS attacks. Fortunately, through our experiments, our mechanism can integrate with other technologies without any conflict to provide a total multi-tier solution.

As mentioned in the section 5, because the *n*_*i*_ and $\bar{d_{i}}$ are objective, for improving the performance of the solution, (i) the short entry idle_timeout, *e*_*s*_, should be small as much as possible; and (ii) the normal idle_timeout, *e*_*n*_, should be large as much as possible while maintaining some transmission constraints. Therefore optimizing these parameters to achieve an effective performance is one of our future research direction.

Moreover, recently, there are a lot of research results proposed in order to address the scalability issues in SDN. Due to the fact that SDN controller is centralized for the SDN, we need various algorithms in order to achieve high performance and scalability in large-scale networks (e.g., convergent networks or data center networks) including clustering algorithms, distributed controlling techniques, NFV integrations. In this paper, we intend to propose an effective approach which can protect the SDN network against DoS attacks where the SDN network has already applied above solutions to ensure scalability feature. However, in order to extend the contributions of our proposed solution, the scalability can also be introduced as main future research topics.

## 7 Conclusion

The proposed ASP mechanism in this paper efficiently protects SDN-based convergent networks against DoS attacks. The mechanism uses a Probabilistic History-based IP Filtering technique for detecting user types and then applies the ASP mechanism along with suitable policies for preventing the effects of DoS attacks. The proposed ASP mechanism is well suited against DoS attacks and thus reduces a significant volume of DDoS attacks. In order to verify the performance of our proposed ASP mechanism, intensive performance evaluation has been conducted and the results show that the average number of flow entries in the switch and the average number of request packets arriving at the ASP-enabled controller are reduced up to 38.23% and 36.17%, respectively. In future research, we will mitigate the aforementioned limitations in Subsection 6.4 and expand the proposed solution to apply in other areas such as application servers and data center.

## Supporting Information

S1 Dataset A part of CAIDA Anonymized Internet Traces Dataset. (XLSX)
